# Editorial: The Silent Cry: How to Turn Translational Medicine Towards Patients and Unmet Medical Needs

**DOI:** 10.3389/fmed.2020.00069

**Published:** 2020-03-04

**Authors:** Manuela Battaglia, Salvatore Albani, Berent Prakken, Norman D. Rosenblum

**Affiliations:** ^1^Telethon Foundation, Rome, Italy; ^2^Duke-NUS Medical School, Singapore, Singapore; ^3^University Medical Center Utrecht, Utrecht University, Utrecht, Netherlands; ^4^Department of Paediatrics, Hospital for Sick Children, Toronto, ON, Canada

**Keywords:** translational research, translational medicine (TM), unmet medical needs, patient engagement in healthcare, interdisciplinary

Translational Medicine encompasses the continuum of activities that extend from the conception of an idea all the way till the development of new therapies and diagnostics for the benefit of patients. The purpose of this Research Topic “The Silent Cry: How to Turn Translational Medicine Towards Patients and Unmet Medical Needs” is to describe a new collaborative model of performing and teaching translational medicine revolving around an understanding of patient and societal needs, rather than on an exceptional idea desperately looking for a market need.

The translational medicine journey should ideally start with patients as engaged collaborators (Battaglia et al.), and continue to involve a myriad of stakeholders from basic scientists and physician scientists to intellectual property attorneys, regulatory professionals, and funders—including industry, as the innovation moves from “the bench to the bedside” (Tabori et al.). However, practically all translational medicine programmes to date have been driven by physician scientists and/or basic scientists with a personal passion, often working with minimal training and support, as their career-path doesn't align with that prescribed for either profession (van Dijk et al.), i.e., treating patients or publishing high impact papers. The dearth of patient inputs and of appropriate team-based problem-solving leads to potential medical solutions falling into unsurmountable “valleys of death,” eventually resulting in wastage of talent, research, and funding.

The only way forward is to truly revolutionize translational medicine by making available appropriate education and support networks (Gohar, Gohar et al.). Training for translational medicine professionals requires not only the scientific and clinical skills that are currently taught in graduate or medical schools, but the soft skills required for creating an effective interface with society and patients as the primary stakeholders of an existing unmet need, as well as the managerial skills to orchestrate collaborations for the regulatory and business considerations (Gohar, Maschmeyer et al.). The curriculum should thus teach critical reflection and collaboration (Clay et al.), and include a “hidden” portion which teaches one to appreciate others' viewpoints as well as hones one's own communication skills (Foty et al.). The focus of both the training programs and environment in which translational medicine professionals work should be inter-disciplinary and focused on creating societal impact, rather than viewing publications as the last judgement (Kools et al.).

Efforts are underway to drive Translational Medicine toward this ideal (https://www.ncbi.nlm.nih.gov/pmc/articles/PMC6419973/). Training programmes like the one run by Eureka Institute for Translational Medicine are currently providing the necessary education to a select cohort of professionals (Weggemans et al.). However, a change in how translational medicine is done and perceived by society will involve a change in thinking at a personal and institutional level. In today's data-driven world, both hospital systems and translational medicine professionals need to understand the considerations and implications of handling and mining vast patient datasets, and strive for a synergism between hypothesis-driven and data-driven experimentation to attain the best possible outcomes (Hulsen et al.). Translational Medicine professionals also need to be more actively engaged with social media to make sure advances are communicated with society accurately and to increase engagement with their stakeholders (Dijkstra et al.). Though basic scientists and physician scientists realize the potential of social media in making connections, for example between the innovators and physicians who run clinical trials, currently there is a lack of its practical use within the community (Sandalova et al.). Moreover, there is a need for creative problem solving during the translational medicine process (Goeltzenleuchter et al.), which will develop over time as the ecology matures and the various stakeholders truly appreciate each other's contributions and priorities while working together. To further strengthen team work and bring in different view points, there is also a need to create gender equity in the field, which can only be achieved by providing better societal support, hiring opportunities and mentoring for women in STEM (Bots et al.).

Despite the long road ahead, there are a number of successful translational medicine programmes that are already underway in a variety of disease areas. Immunomics in pediatric rheumatic disease has seen a number of advances in genomics, transcriptomics, epigenomics, proteomics, and cytomics—all unearthing new prognostic biomarkers and creating avenues for creating new therapies (Tay et al.). There are also trials underway to treat Type I Diabetes with immunotherapy (Coppieters and von Herrath), and currently innovative strategies such as combining immunotherapy with agents that promote Beta-cell survival are being tested. In the field of vaccine development, Controlled Human Infections (CHI) and question-based clinical development approaches are providing solutions to make vaccines more cost-effective and efficacious (Roestenberg et al.; Roestenberg et al.). The field of cognitive medicine is also progressing rapidly with advances in technologies like Diffusion Tensor Imaging (Lock et al.).

In summary, Translational Medicine is continuously evolving and as the field attracts more talented professionals with structured funding and career pathways available for their success, faster and better medical solutions will reach patients in need sooner. To fuel this Translational Medicine discipline, both physician scientists and basic scientists with a focus on patient-oriented research outcomes are needed. Early exposure to interdisciplinary environments and an organized institutional framework, including a dedicated program for translational medicine with accessible mentors is crucial. Reconsideration of the publication system and strategies for including important stakeholders throughout the process will put translational medicine advances in societal context, driving innovation in both directions.

## Author Contributions

All authors listed have made a substantial, direct and intellectual contribution to the work, and approved it for publication.

### Conflict of Interest

The authors declare that the research was conducted in the absence of any commercial or financial relationships that could be construed as a potential conflict of interest.

